# Investigating the Microchannel Architectures Inside the Subchondral Bone in Relation to Estimated Hip Reaction Forces on the Human Femoral Head

**DOI:** 10.1007/s00223-021-00864-x

**Published:** 2021-05-22

**Authors:** Shahed Taheri, Takashi Yoshida, Kai O. Böker, Robert H. Foerster, Lina Jochim, Anna Lena Flux, Birgit Grosskopf, Wolfgang Lehmann, Arndt Friedrich Schilling

**Affiliations:** 1 Department of Trauma Surgery, Orthopaedic Surgery and Plastic Surgery, University Medical Center Göttingen, Göttingen, Germany; 2 Department of Historical Anthropology and Human Ecology, University of Göttingen Johann-Friedrich-Blumenbach, Institute for Zoology & Anthropology, Göttingen, Germany

**Keywords:** Bone microarchitecture, Cartilage-bone marrow microchannel connectors, Human femoral head, Subchondral bone, Gait analysis, Bone micro-CT

## Abstract

The interplay between articular cartilage (AC) and subchondral bone (SB) plays a pivotal role in cartilage homeostasis and functionality. As direct connective pathways between the two are poorly understood, we examined the location-dependent characteristics of the 3D microchannel network within the SB that connects the basal cartilage layer to the bone marrow (i.e. cartilage-bone marrow microchannel connectors; CMMC). 43 measuring points were defined on five human cadaveric femoral heads with no signs of osteoarthritis (OA) (age ≤ 60), and cartilage-bone cylinders with diameters of 2.00 mm were extracted for high-resolution scanning (*n* = 215). The micro-CT data were categorized into three groups (load-bearing region: LBR, *n* = 60; non-load-bearing region: NLBR, *n* = 60; and the peripheral rim: PR, *n* = 95) based on a gait analysis estimation of the joint reaction force (young, healthy cohort with no signs of OA). At the AC-SB interface, the number of CMMC in the LBR was 1.8 times and 2.2 times higher compared to the NLBR, and the PR, respectively. On the other hand, the median Feret size of the CMMC were smallest in the LBR (55.2 µm) and increased in the NLBR (73.5 µm; *p* = 0.043) and the PR (89.1 µm; *p* = 0.043). AC thickness was positively associated with SB thickness (Pearson's *r* = 0.48; *p* < 1e-13), CMMC number. (*r* = 0.46; *p* < 1e-11), and circularity index (*r* = 0.61; *p* < 1e-38). In conclusion, our data suggest that regional differences in the microchannel architecture of SB might reflect regional differences in loading.

**Supplementary Information:**

The online version contains supplementary material available at 10.1007/s00223-021-00864-x.

## Introduction

It is widely accepted that osteoarthritis (OA) is a disease of the whole joint that involves articular cartilage (AC), subchondral bone (SB), as well as synovium, menisci, adjacent ligaments, and the surrounding soft tissues [1–3]. The interplay between AC and SB is known to be crucial in understanding the pathogenesis of OA, making osteochondral unit, and in particular SB, a focal point of OA research [4–8].

Even though mature SB was long considered to be a closed end-plate with assumed impermeability of the calcified cartilage (CC) layer [9–12], several reports have suggested that the basal layer of the AC is connected to the medullary cavity via some breaks or defects. We have recently shown in a bovine model that cartilage and bone marrow are connected by a network of microchannels (i.e. cartilage-bone marrow microchannel connectors; CMMC), which exhibited age-related microstructural changes [13]. Direct contact of the basal cartilage layer with deeper trabecular bone has been reported in adult non-pathological human femoral heads [14–16] as well as mature human knee joints [17–19], while components such as blood vessels, osteochondral tissues, parallel lamellar bone, woven bone fibers, fat, and other unidentified soft tissues were observed underneath such contacts [16]. Moreover, SB porosity has been detected in end-stage knee OA of patients with different joint alignments, showing region-specific associations with the subchondral bone plate thickness [20]. This raises the question of the physiological and pathophysiological significance of these microchannel connectors for the overlying cartilage.

These perforations have been either categorized based on their morphology [21]; vascularity [14, 15]; or a combination of size-based, component-based, and topographical features [16–18]. As a result, the morphological differences have been interpreted differently. For example, the long finger‐like microchannels have been described either as a marker related to the health-degree of the overlying AC [22], or a region-specific feature of the weight-bearing areas, irrespective of the health-state of the joint [17, 18]. Similarly, large vascular void spaces have been described both as pathological features that are exacerbated with OA progression [22], or as non-pathological occurrences in normal (i.e. intact cartilage) human joints [18], as well as various healthy animal models that are generally detected at the non-weight-bearing areas [17, 21]. Other disputed questions are if the perforations cross through the calcified cartilage layer [23, 24], and whether they are rare or frequent occurrences in old populations [18, 25]. These discrepancies so far prevented a unified hypothesis regarding the function(s) of the SB perforations and their potential role as diagnostic and therapeutic targets [23, 25]. Possible reasons for this contradictory evidence and subsequent research gap could be (1) the lack of spatial illustration of the perforations in the so far implemented 2D approaches (i.e. scanning electron microscopy, photomicrography) and (2) disregarding variances in the tissue architecture that would give different results, depending on the studied location.

It is generally accepted that bone is highly adaptive to habitual loading, regulating its structure according to components of its loading regime and mechanical environment such as strain magnitude, rate, frequency, distribution and deformation mode [26]. Thus, it is conceivable that the microstructure of different areas of the SB may vary according to the repetitive local loading of the joint. In this regard, combining a gait analysis-based reaction force estimation on the joint to determine probable loaded areas, and a micro-CT technique for mapping of the SB microarchitecture could be a useful approach.

Therefore, the aims of this study were (1) to visualize the spatial architecture of the cartilage-bone marrow microchannel connectors in regions of healthy human femoral heads that are presumably subjected to different regional loading. And (2) to quantitatively map the location-dependent differences of the SB microarchitecture in the healthy human femoral head by defining new canal metrics. It is hypothesized that the characteristics and the spatial distribution of these CMMC might be influenced by local joint forces, which were derived from the force estimations during self-paced walking of a separate healthy cohort with no signs of OA. Specifically, we postulate that the local density of CMMC is higher in areas of the joint that are possibly subjected to repetitive local loading, and that incorporation of CMMC in the model of osteochondral junction may contribute to a better understanding of joint physiology and pathology. This preliminary study may allow for the formulation of hypotheses that can be used as foundation for future studies in OA research, and revitalize discussions on possible significance of the SB microchannel network.

## Subjects and Methods

### Biopsy and Preparation of Human Bone Specimens

**Fig. 1:**
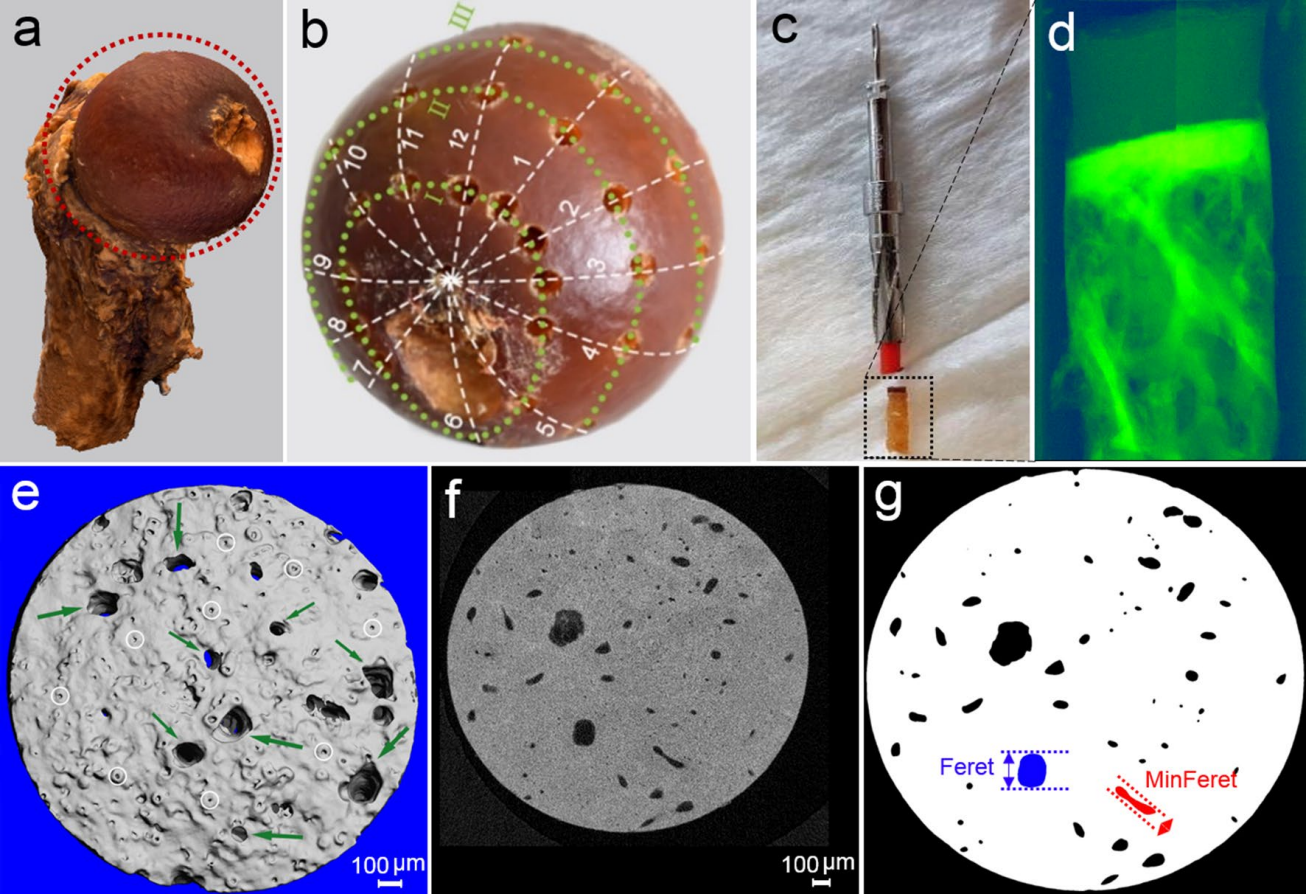
A Schematic overview of the experimental workflow. **a** 3D model of a formalin-fixed healthy human femur. **b** 43 measuring points were defined as the intersections of twelve concentric lines and four parallel parasagittal planes (labeled with Roman numerals). **c** 2.00-mm cylindrical specimens were extracted from each measuring point by a trephine burr, and scanned. **d** The scout view of an exemplary sample, showing the low-density articular cartilage and the high-density subchondral bone. **e** The superior view of a 3D-reconstructed sample showed cartilage-bone marrow microchannel connectors (CMMC) at the top surface of the SB (marked by green arrows), which are distinguished from surface imperfections and craters (white circles). The grey-scale images **f** were binarized **g** for quantification. The schematic description of the CMMC size metrics (i.e. maximum and caliper diameter, Feret and MinFeret, respectively) are presented for two exemplary microchannels as the lengths between the parallel dotted lines

Anonymous cadaveric human femurs were provided by the anatomical gift program of the Medizinische Hochschule Hannover (MHH). All donors had given their informed consent to the donation of their corpses for research purposes. Femurs were embalmed in a 10% neutral buffered formalin solution, put individually in plastic bags, and were stored in sealed containers at + 4 °C. Due to the anonymous nature of the MHH gift program, the age, sex, medical history, and OA status of each donor were unknown. Hence, the femurs were independently graded by three orthopaedic surgeons according to the Outerbridge classification system that describes AC lesions [27]. Samples with a mean Outerbridge grade < 0.5 that did not show macroscopically visible AC damage or other signs of OA were considered healthy and shortlisted for selection. This criterion can be interpreted of a case where at least two surgeons allocated a grade 0 (normal cartilage) to a specific donor, while the other surgeon assigned a maximum grade of 1 (cartilage with softening and swelling). Additionally, DNA were extracted using a protocol for formalin-fixed materials [28], and the sex was subsequently determined by a PCR-based method. The fact that the cohort should be homogeneous in terms of conditions of the joint (OA grade), sex, and the anatomical position of the organ (right vs. left), restricted us to select right-leg female femurs. Hence, five healthy samples that showed female-specific bands at 163 bp (see Online Resource 1) were selected for measurements and analysis (Fig. 1a). Exclusion criteria were artefacts (e.g. iatrogenic erosions, tears, or elevations) and striking deformities (i.e. coxa vara, coxa valga).

By assessing the structural elements of the cortical bone such as primary and secondary osteons, Haversian canals, endosteal and periosteal lamellar bone, and areas of bone resorption, whose quantity or proportion undergo age-dependent changes, the biologicals ages at death can be determined (see Online Resource 2). All samples were identified to have biological ages of 40 to 60 years at death, with the exception of one case, for which the age could not be determined more precisely than its general category (older than subadult, i.e. older than eighteen years of age).

In order to employ the maximum resolution of the micro-CT device, (2.00-mm diameter) cartilage-bone cylinders were extracted by a trephine burr from 43 measuring points on each sample (Fig. 1b, c). They were systematically defined as the intersections of two sets of geometric shapes that were drawn on the femoral heads: 1) twelve concentric lines, each separated by a 30° interval, starting from the center of the femoral head and ending on the neck junction, and 2) four parallel parasagittal planes, which divided the arc between the center and the neck junctions into four equally-spaced regions. The center of the femoral head was defined as where the long axis and the coronal plane intersect on the surface of the femoral head. The location of each set of measuring points was standardized using a template grid, taking into account the normalized size of each joint. This division system is partially inspired by the work of Turmezei et al. [29].

### Acquisition and Analysis of Micro-CT Data

Bone extracts were washed repeatedly with 70% ethanol to remove potential bone debris, placed vertically into sample holders, and were scanned in 70% ethanol using a high-definition micro-CT (µCT 50, SCANCO Medical AG, Switzerland) with the following settings: voxel size = 1.2 µm, source voltage = 90 kVp, intensity = 88 µA, integration time = 1500 ms, projections/180° = 1500, and 0.5 mm aluminum filter, scanning time ≈ 3.5 h, reconstructed image dimensio*n* = 3400 × 3400 (pix), data size ≈ 15 GB. The voxel size of 1.2 µm was determined based on the reports of smallest type of SB canals being in the 10–45 µm range [17, 18]. The high-resolution scans were then used for 3D reconstruction and quantification of the SB microstructure.

Since the density of AC and 70% ethanol solution appear to be in the same vicinity in the micro-CT images, AC cannot be distinguished and segmented when the biopsies are scanned in 70% ethanol. Nonetheless, AC can be detected as a lesser-dense object and segmented properly when scanned dry, even without using a contrast agent (Fig. 1d). It is known that dehydration can result in a nonlinear cartilage tissue shrinkage [30]. Thus, in order to avoid shrinkage of the AC in dry conditions, the time that the biopsy was kept in the dry condition was reduced by performing fast-secondary scans (< 30 min) at a voxel size of 14.8 µm for each of the measuring points (reconstructed image dimensio*n* = 1024 × 1024 (pix), data size ≈ 1 GB, projections/180° = 500, all other setting parameters were identical to the scanning performed at 1.2 µm). Using a 3D segmentation script of the Scanco’s OpenVMS software for three volume of interests, the cartilage was distinguished and segmented from the air and bone (air: -500 – -140 mg HA/cm^3^, cartilage: -140–600 mg HA/cm^3^, bone: 600–3000 mg HA/cm^3^, Sigma: 2, Support: 4). The segmented models were then imported to Fiji (National Institutes of Health, Maryland, USA [31]) to measure AC and SB thicknesses. For each sample, the measurements were conducted at 60 fixed locations within three equally-spaced cross-sections of the model to obtain a mean value.

For quantification of the CMMC, the immediate intersection of AC and SB (tidemark) must be in a uniformly horizontal plane (i.e. XY). Thus, the deviations above 1-degree angles from the XY plane were aligned using a lossless rotation command (Scanco’s image processing language; IPL). Binarized layers were then created as TIFF stacks, and imported into Fiji for further analysis (Fig. 1g). The volume of interest (VOI) was a cylinder with a diameter of 1800 µm and height of 400 µm that spanned from the tidemark to the subarticular region, encompassing the entire SB. The outer 100-µm rim of the samples were excluded to avoid incorporating potential bone debris and other artifacts. The height of the VOI was selected based on the thickest measured subchondral bone plate.

CMMC number was defined as the number of microchannels per mm^2^. In each transverse plane of binarized images, the CMMC number was computed as the number of individual structures with a tracking value of zero, divided by the area of the plane. Since the CMMC are not always symmetrical, their size was quantified by the maximum caliper diameter (Feret), which is defined as the longest distance between any two points along the selection boundary. Likewise, the minimum caliper diameter (MinFeret) was measured to calculate the shortest distance that can be measured by the CMMC boundary (see Fig. 1g for schematic descriptions of Feret and MinFeret). The morphology of the CMMC was quantified by the circularity index (Circ. = 4π(area/perimeter^2)). Circ. of 1.0 indicates a perfect circle, while Circ. closer to 0.0 indicates an increasingly elongated polygon (e.g. the blue and the red microchannels illustrated in Fig. 1g have circularity indices of 0.868 and 0.344, respectively).

An ImageJ Macro language algorithm was developed that could calculate the previously-defined metrics of the CMMC (i.e. CMMC number, Feret, MinFeret, and Circ.) in a layer-by-layer basis using Fiji (National Institutes of Health, Maryland, USA). Discontinuous pores below the Feret size of 7 µm were categorized as craters, associated with the calcification of the cartilage [17], and were consequently filtered out., To categorize the output of the algorithm (i.e. CMMC metrics) into groups that represent different areas on the femoral head, hip reaction forces were estimated from the gait analysis on a separate healthy cohort, as outlined below. Quantitative analysis for each of the defined CMMC metrics is presented as average value changes versus distance from the tidemark, where profiles in each identified region are means of the corresponding measuring points. The reported values at the AC-SB interface are medians of the five subjects at the uppermost 50 µm of the subchondral bone, which are presented together with corresponding lower and upper quartiles (i.e. MED (Q_1_ to Q_3_)).

### Joint Reaction Force Estimation

It is known that osteoarthritis, as well as the angulation within the shaft of the long bones (i.e. Valgus and Varus alignments) can influence the gait analysis of individuals [32, 33]. Therefore, it is presumed that in the absence of such factors, the joint forces of non-pathological subjects with no pre-clinical signs of OA that have normal joint alignment are comparable during normal repetitive activities such as walking. Hence, the joint reaction force of a young healthy cohort at the right hip was estimated in order to identify possible load mapping on the femoral head, and to use this as a physiological reference to categorize the micro-CT data. This was performed using an open-source biomechanical analysis software (OpenSim [34]), a compatible musculoskeletal model (Gait 2392), which included 23 degrees of freedom and 92 musculotendon units, and experimental data from eight able-bodied men (age: 25 ± 3 years, height: 188 ± 3 cm, weight: 86 ± 8 kg). This represented a young non-pathological cohort with no signs of OA, which was nonetheless different from the healthy donors used for the micro-CT analysis. In the musculoskeletal model, the hip joint was a dimensionless ball-and-socket joint, rather than an anatomically-faithful three-dimensional contact area. Before the experiment, the participants gave their written informed consent, and the experimental protocol had been approved by the local ethics board at the University Medical Center Göttingen, Göttingen, Germany (application number: 14/6/17). Each participant walked at a self-selected comfortable pace and completed four trials. During each trial, whole-body kinematics and ground reaction forces were recorded. To record body kinematics, a motion capture system with twelve infrared cameras (Bonita B12, Vicon MX, Vicon Motion Systems Ltd, UK) and retroreflective markers were used. The markers were placed over bony landmarks as well as shanks and thighs in clusters of three [35]. Marker trajectories were sampled at 200 Hz. To record ground reaction forces, we used two identical force plates (9287A, Kistler Group, CH), each of which recorded the forces on one foot at 1000 Hz. For each trial, the time window for analysis began with the left toe off and ended with the subsequent right heel contact. The measured marker trajectories and ground reaction forces were low-pass filtered at 6 Hz. Using the marker trajectories from a separate static trial, a unique musculoskeletal model was generated for each participant using the Scaling function of OpenSim. The scaled model was used in a sequence of analyses that used the Inverse Kinematics, Residual Reduction Analysis, Computed Muscle Control, and Joint Reactions Analysis functions of OpenSim, the last of which estimated the reaction force at the hip joint, exerted by the femoral head. The reaction force was normalized to the body weight of each participant and expressed in a local reference frame, which was fixed to the center of the femoral head. For each participant, we calculated the average force vector over the four trials, and these intra-participant averages were used to calculate the group-average force vector. Subsequently, we visualized the intersection between the group-average force vector and a unit sphere, which represented the femoral head. The tracing of this intersection over time showed how the hip reaction force was oriented during walking.

**Fig. 2:**
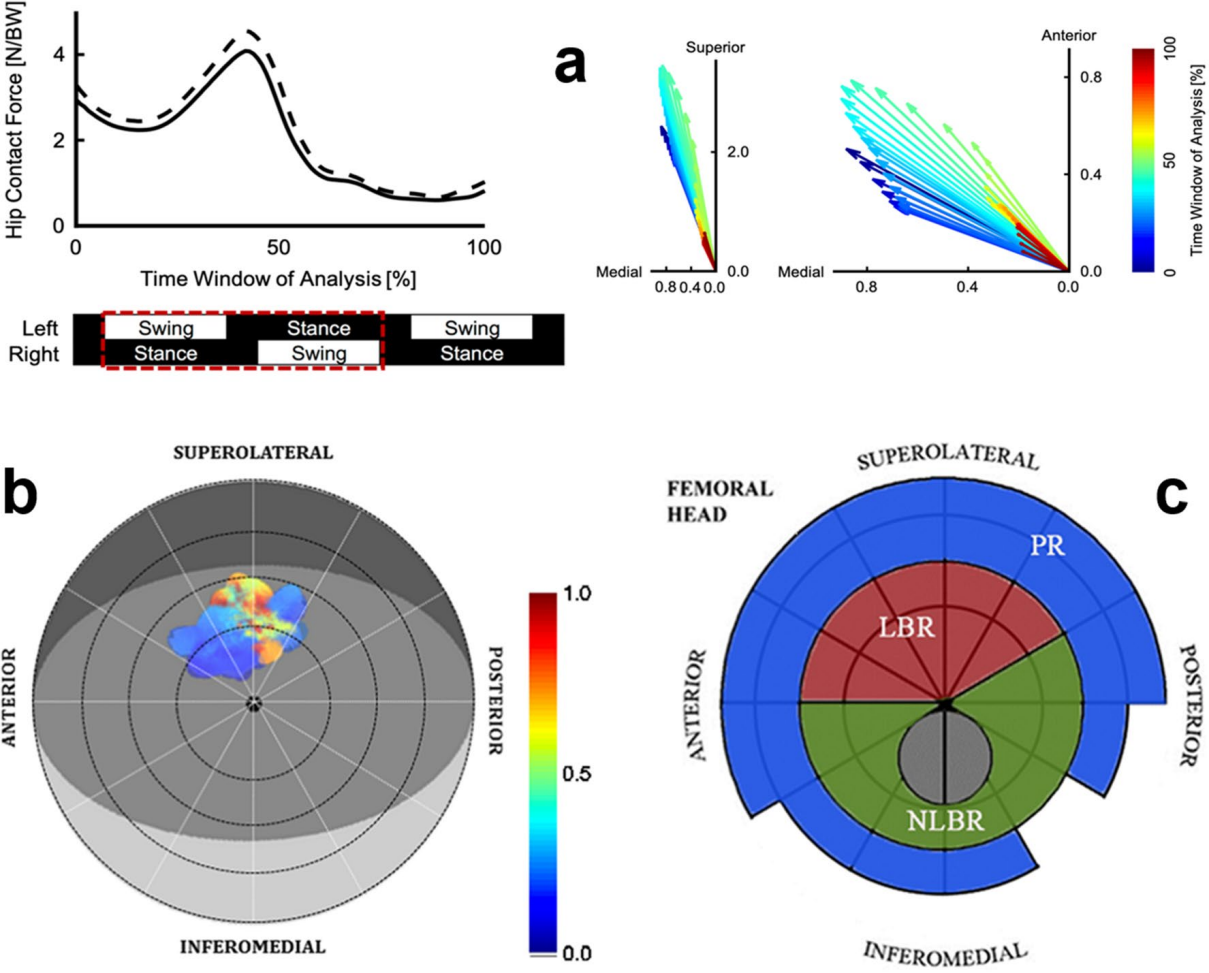
Joint reaction force at the right hip. **a** Magnitude [N/BW] and orientation of the estimated hip reaction force throughout the time window of analysis, which starts at the left toe off and ends at the subsequent right heel contact (group average). The solid line is the inter-participant mean, and the dashed line indicates the inter-participant standard deviation. Color of each arrow indicates the temporal sequence. **b** The tracing of the intersection between the group-average normalized hip reaction force vector and a unit sphere, which represents the femoral head, during the time window of analysis. Each instant of intersection is plotted as a circle with a non-zero diameter for easier visualization. The unit sphere is viewed at azimuth of -15° (the average anteversion angle) and elevation of 50°, with the origin of the reference frame at the center of the sphere (azimuth and elevation of 0° shows the sagittal plane from the medial side. **c** Consequently, probable loading areas are distinguished and color-coded: load-bearing region (LBR; red), non-load-bearing region (NLBR; green), and the peripheral rim (PR; blue). The gray circle within the NLBR is the fovea capitis. The asymmetry in the inferomedial part of the PR is due to the inherent extension of the neck close to the fovea

Figure 2a shows the estimated magnitude and orientation of the right hip reaction force throughout the time window of analysis, which started at the left toe off and ended at the subsequent right heel contact. The hip reaction force peaked during push-off in the superoanteromedial direction, had a large superior component, and always pointed in the medial direction. Figure 2b shows the tracing of the intersection between the group-average hip reaction force and a unit sphere, which represents the femoral head. This tracing shows how the hip reaction force is oriented during walking. Based on this, the area identified as probable load-bearing region (LBR) of the joint was recognized in accordance with the superimposed mapping, as well as previously reported load-bearing areas in normal adult hip joints under conditions typical of walking [16]. The remaining regions of the femoral head were accordingly categorized into probable non-load-bearing region (NLBR), and the peripheral rim (PR; Fig. 2c). The NLBR was located centrally, mostly in the inferior half of the femoral head, while the PR points were located at the two outermost parasagittal planes close to the neck junction. The recognition of the peripheral rim as a distinct regional entity was not made by the joint reaction forces alone, but was taken into consideration by other reports regarding differences in the SB microstructure between the superocentral contact areas and the peripheral rim close to the femoral neck junction [14, 18]. Thus, the CMMC metrics are reported for the probable LBR (12 measuring points per subject, out of the total 43), NLBR (12 measuring points per subject), and the PR (19 measuring points per subject).

### Histological Methods

In order to validate earlier observations of the CMMC from the micro-CT imaging, toluidine blue staining was performed on decalcified biopsies according to a standard protocol [36]. Samples from each identified area were randomly selected and decalcified in ethylenediaminetetraacetic acid (EDTA), where they were immersed in 5 ml of 20% EDTA/Tris–HCl, pH 7.4, and gently agitated for 72 h at 4 °C. They were dehydrated in ascending concentrations of ethanol (70–100%; each concentration for 3 days) after washing repeatedly with water, embedded in paraffin wax, and then sectioned coronally (5 μm). Finally, the samples were stained in 0.05% toluidine blue for 10 min after paraffin removal, sequentially dehydrated in ethanol (79–100%), and then cleared in xylene.

## Statistical Analysis

To estimate the sample size for this study, a priori power analysis was performed using the G*Power software (Heinrich-Heine University Düsseldorf, [37]) assuming a standard deviation of 25% and a power of 80%. The effect size (Cohen’s d = 0.37) were calculated from a pilot study based on the estimated mean values of the CMMC number (8 1/mm^2^ vs. 4 1/mm^2^) and the Feret size of the microchannels (50 µm vs. 80 µm) in the LBR and NLBR regions. Hence, the projected sample size needed to assure that the CMMC metrics within the identified regions differ with a probability of error of α ≤ 0.05 was approximately five (*n* = 5) according to this estimation. As the subjects are examined under all of the levels of a within-subject factor (i.e. identified regions), repeated measures analysis has to be implemented. Hence, a Friedman test was conducted for one-way repeated measures analysis of the variances in the group mean ranks, taking into account the loading region (i.e. LBR, NLBR, and PR) as the independent, within-subjects factor. When significant differences were observed, a Wilcoxon signed-rank test was administered for comparisons in pairs. As the critical value of the Wilcoxon signed-rank test at the level of 0.05 is zero, a significant difference between group pairs is only attainable when all five subjects have either positive or negative ranks, in which case the *p*-value is 0.043. Hence, the measured p-value in this study is highly conservative, and merely shows the significant differences, and not the level of significance between the groups. For the same reason, multiple-comparison adjustment could result in type II error (i.e. false-negatives), and therefore, has not been conducted [38]. Correlation analysis was achieved with Pearson test to study possible relationships between the cartilage thickness and other variables of the subchondral bone microstructure for all the biopsies across the entire femoral head (*n* = 215). The best linear fit was plotted, with a simultaneous ANOVA to calculate the *p* values. Throughout the manuscript, the median values of each group are given including the lower and upper quartiles (i.e. MED (Q_1_ to Q_3_)). *p* values < 0.05 were considered statistically significant. Statistical evaluation was carried out using IBM SPSS Statistics (Version 25, Armonk, NY: IBM Corp).

## Results

### Subchondral Trabecular Bone is Connected to the Basal Cartilage via a Complex Microchannel Network

**Fig. 3:**
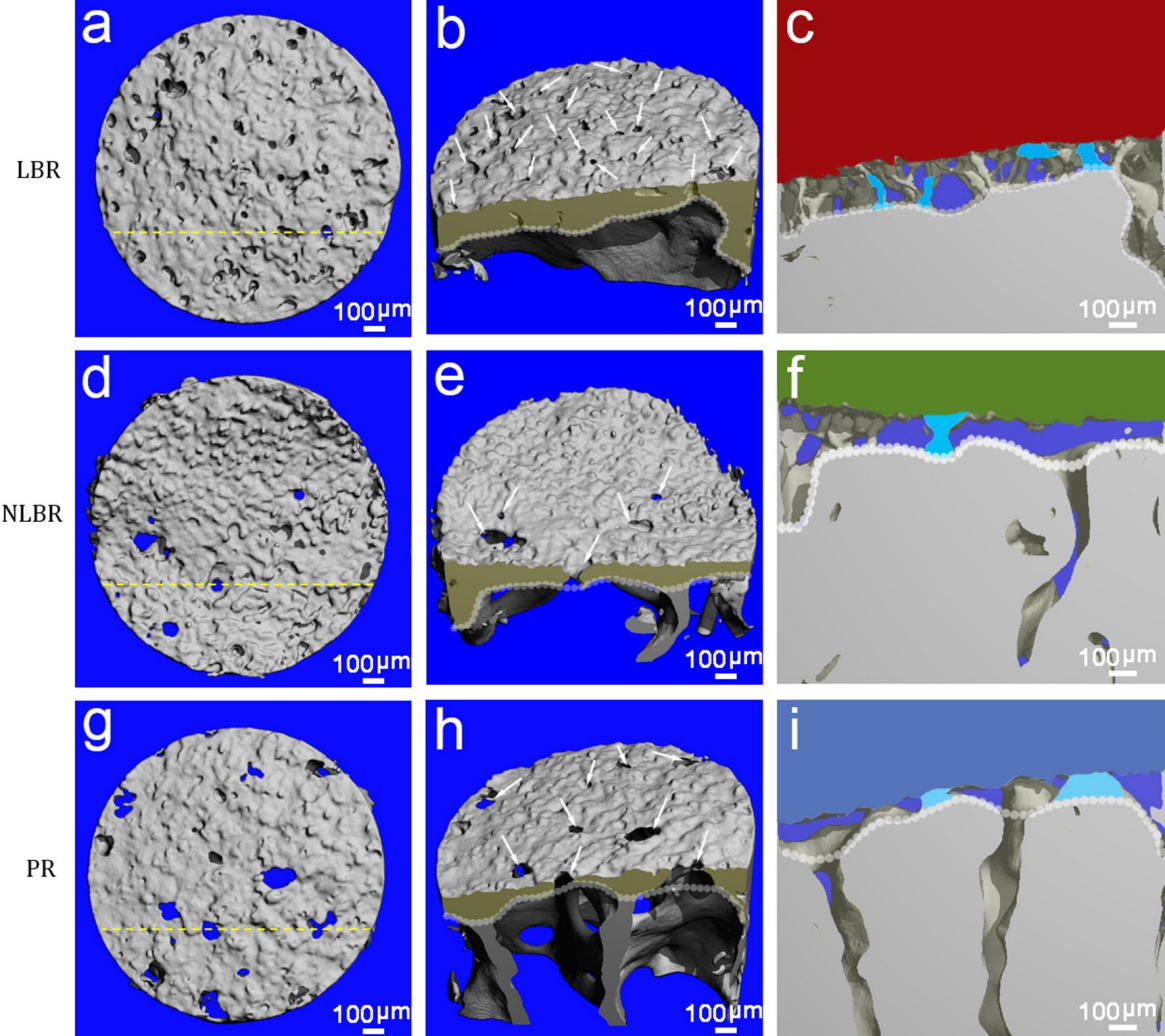
The 3D representation of the CMMC of the SB in healthy human femoral heads. **a** The superior view of a typical LBR sample revealed abundant microchannels reaching to the most superficial surface of the SB. **b** The cross-section of the same sample at an arbitrary plane (yellow dotted line in **a**), showed individual microchannels (white arrows) passing through the SB (dark yellow). **c** In the inverted, "negative" model of the exact cross-section, cartilage of the LBR is shown in red, osseous structures in transparent, and the CMMC in grey. Individual microchannels cut by the cross-section are shown in cyan. The superior, cross-section, and the negative views of the NLBR and the PR are illustrated in (**d-f)** and (**g-i**,) where cartilage is depicted in green (**f**) and blue (**i**), respectively. The white dotted lines in the images of the central and the right columns signify the lower margin of the subchondral bone plate

The 3D-reconstructed images of the SB confirmed the presence of a porous microstructure that directly connected the AC-SB interface to the trabecular spongiosa via cartilage-bone marrow microchannel connectors (CMMC). We found a distinct distribution pattern for the CMMC based on their location on the joint. In regions that are probably load-bearing (i.e. LBR), many small channels reached to the most superficial surface of the subchondral bone and were in direct contact with the basal cartilage (Fig. 3a, b). The inverted 3D-representation of this region showed the continuous nature of these microchannels throughout the entire subchondral bone (Fig. 3c). On the other hand, the NLBR was mainly characterized by a sporadic CMMC distribution that had bigger contact areas with the basal portion of the AC in comparison with the LBR (Fig. 3d–f). At the PR of the femoral head, the microarchitecture of the subchondral bone mainly comprised of non-circular CMMC with the highest channel sizes observed (Fig. 3g–i).

### CMMC are Abundant, Small, and Circular in the LBR While Less Frequent, Large, and Elongated in the Peripheral Rim of the Femoral Head

**Fig. 4:**
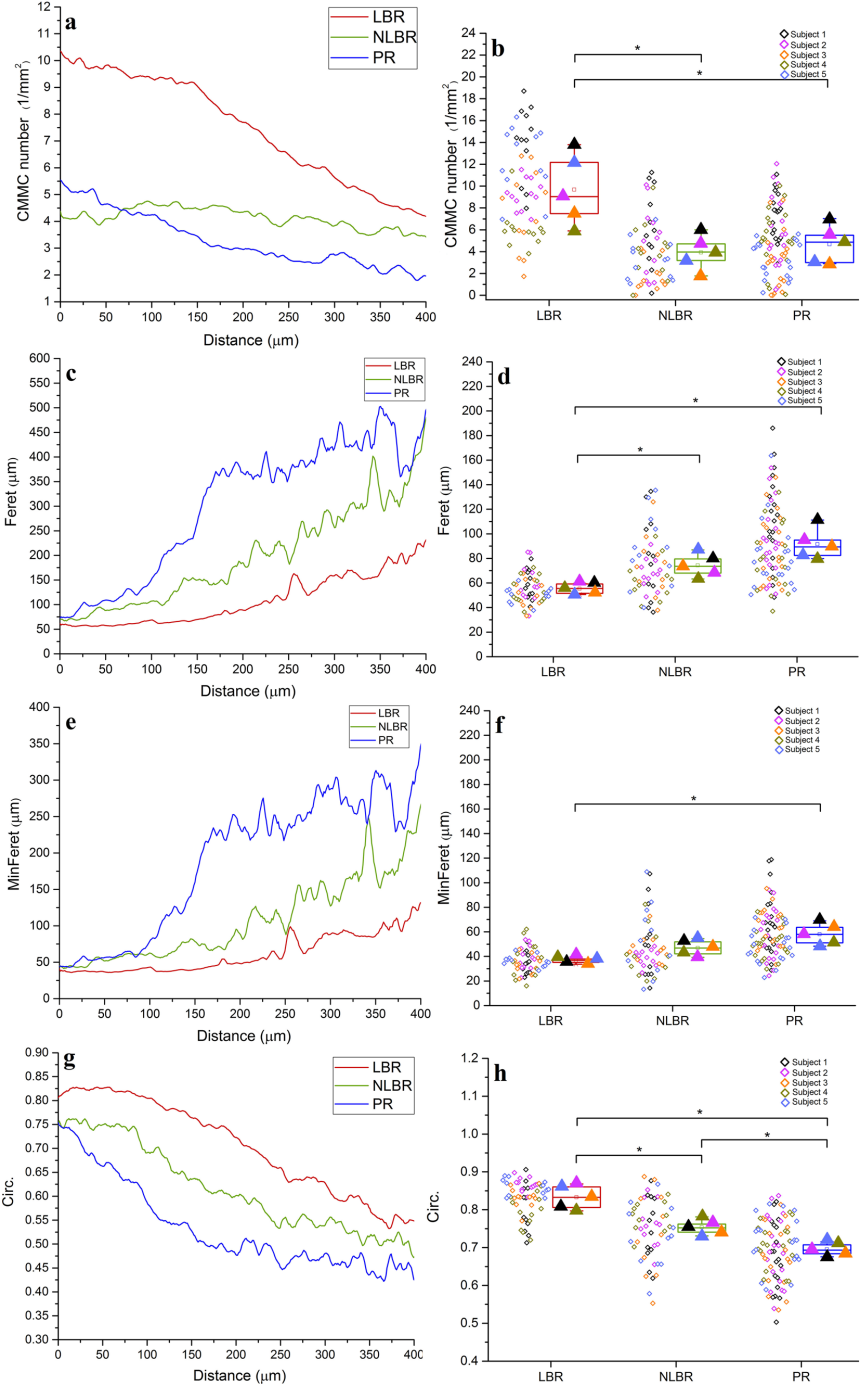
Quantitative characterization of the cartilage-bone marrow microchannel connectors. The profile of the CMMC number, Feret, MinFeret, and Circ. vs. distance from the tidemark for different identified regions are illustrated in (**a**, **c**, **e**, and **g**), respectively. In the uppermost 50 µm of the subchondral bone, the changes of the CMMC number (**b**) Feret (**d**), MinFeret (**f**), and Circ. (**h**) are shown as boxplots. Each oblique square adjacent to the boxplots represents the mean value of a measuring point, and is color-coded to its corresponding subject. The filled triangles signify the mean values of corresponding subjects in each identified region. *p < 0.05

The profiles of the CMMC number vs. distance from the tidemark are distinct for different regions of the femoral head, with the LBR having consistently a higher number of CMMC compared to the other two regions (Fig. 4a). At deeper distances from the tidemark (> 150 µm), there was a steep decline in the slope, representing the coalescence of the microchannels close to the lower margin of the subchondral bone plate. Beyond the subchondral bone plate (i.e. subchondral trabecular bone), the values do not represent the microchannels of the SB anymore, but the spacings between the trabeculae. The microchannels that cross the calcified cartilage region and are in direct contact with the basal cartilage are particularly interesting due to their potential physiological implications. Hence, the mean CMMC number of each measuring point, and each subject is illustrated in the top 50 µm of the subchondral bone (Fig. 4b). The Friedman test indicated a significant region-based effect (χ^2^(2) = 8.40, *p* = 0.015), where the median CMMC number (1/mm^2^) of the five subjects for the LBR, NLBR, and the PR were 9.21 mm^−2^ (6.87 to 13.15 mm^−2^), 4.12 mm^−2^ (2.64 to 5.51 mm^−2^) and 5.15 mm^−2^ (3.24 to 6.55 mm^−2^), respectively. Follow up pairwise comparisons showed that the LBR had a significantly higher number of microchannels in comparison with the NLBR and the PR (Z = -2.023, *p* = 0.043). No statistically significant difference was observed between the CMMC number of the NLBR and the PR.

The size of the CMMC throughout the subchondral bone was also region-dependent (Fig. 4c-f). In particular, at the upper 50 µm of the subchondral bone, the pairwise comparisons showed that the maximum caliper diameter (Feret) increased as the region changed from the LBR (MED = 55.2 µm, Q_1_ to Q_3_ = 50.8 to 59.2 µm) to the NLBR (MED = 73.5 µm, Q_1_ to Q_3_ = 65.6 to 83.2 µm; *p* = 0.043) and the PR (MED = 89.1 µm, Q_1_ to Q_3_ = 80.9 to 102.8 µm; *p* = 0.043; Fig. 4d). Similar changes were observed for the minimum caliper diameter of the microchannels, as the LBR (MED = 36.4 µm, Q_1_ to Q_3_ = 34.0 to 38.3 µm) showed the smallest MinFeret, while the minimum diameter of the microchannels was significantly larger at the PR (MED = 57. 6 µm, Q_1_ to Q_3_ = 49.3 to 65.9 µm; *p* = 0.043; Fig. 4f). The median MinFeret of the microchannels at the NLBR was 46.3 µm (Q_1_ to Q_3_ = 40.2 to 52.7 µm), which was not statistically different from the LBR and the PR (Z = -1.752, *p* = 0.08).

The morphology of the CMMC revealed clear location-dependent differences as well (Fig. 4g). Close to the tidemark, the Friedman test showed a significant effect for the Circ. of the microchannels, χ^2^(2) = 10.00, *p* = 0.007. The CMMC were relatively circular in the LBR (MED = 0.83, Q_1_ to Q_3_ = 0.80 to 0.86) while they showed a combination of asymmetrical, elongated, and oval shapes as the region changed to the NLBR (MED = 0.75, Q_1_ to Q_3_ = 0.73 to 0.77; *p* = 0.043) and the PR (MED = 0.69, Q_1_ to Q_3_ = 0.67 to 0.71; *p* = 0.043; Fig. 4h). The difference of the median Circ. between the NLBR and the PR was statistically significant (*p* = 0.043).

### The Distribution of AC Thickness and the SB Thickness Overlaps

**Fig. 5:**
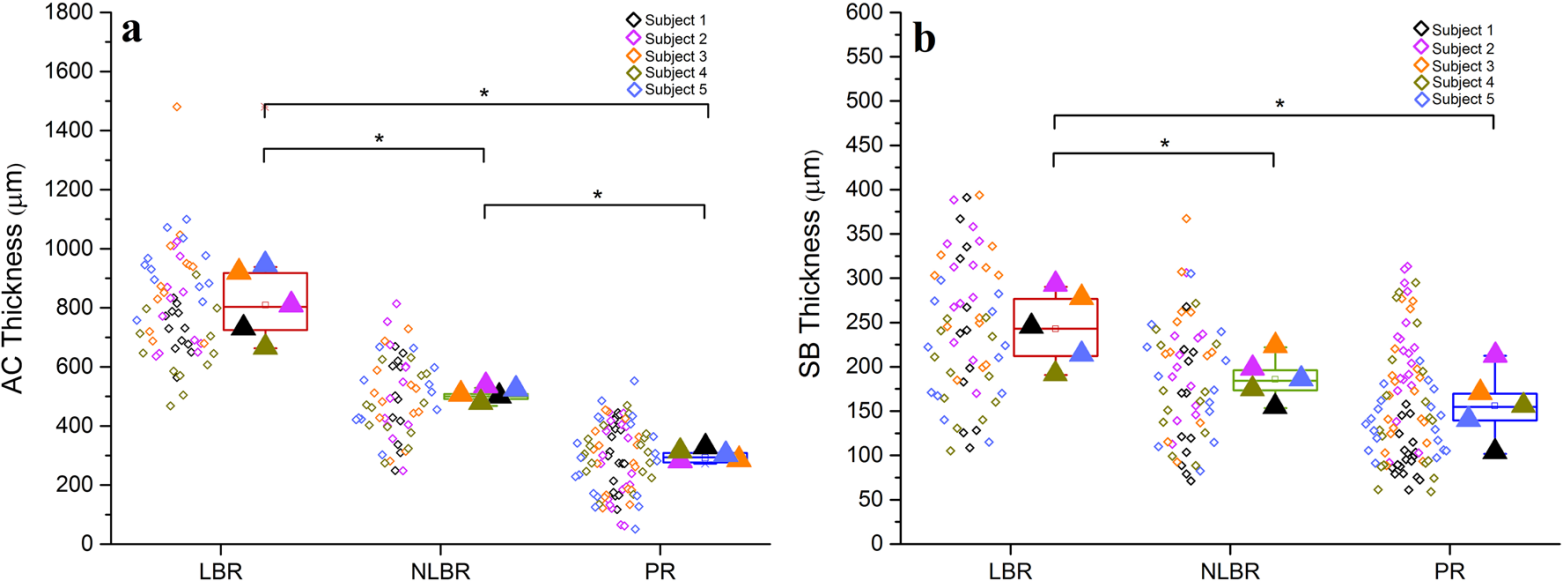
**a** The changes of the AC thickness, and **b** the SB thickness in different identified areas of the femoral head. Each oblique square adjacent to the boxplots represents the mean value of a measuring point, color-coded to its corresponding subject. The filled triangles signify the mean values of corresponding subjects in each potential loading region. The statistical significance was based on a Friedman test followed by Wilcoxon signed-rank tests for pairwise comparison. *p < 0.05

The results of the Friedman test indicated a significant region-based effect for the thickness of the articular cartilage, χ^2^(2) = 10.00, *p* = 0.007. Follow up post hoc analysis with Wilcoxon signed-rank tests revealed that the median (presented as, MED (Q_1_ to Q_3_)) AC thickness for the LBR, NLBR, and the PR were 803.3 µm (693.8 to 928.0 µm), 498.3 µm (477.8 to 516.7 µm) and 294.0 µm (274.5 to 310.8 µm), respectively. In the LBR, the AC thickness was statistically higher compared to the NLBR (Z = -2.023, *p* = 0.043) and the PR (Z = -2.023, *p* = 0.043; Fig. 5a). Similarly, the SB thickness was significantly different in the three identified regions (χ^2^(2) = 8.40, *p* = 0.015), where the median SB thickness was 242.2 µm (200.7 to 282.8 µm), 183.5 µm (162.7 to 208.3 µm) and 154.0 µm (120.0 to 190.5 µm) for the LBR, NLBR, and the PR, respectively. The SB was significantly thicker in the LBR compared to the NLBR and the PR (Z = -2.023, *p* = 0.043; Fig. 5b). When comparing the NLBR and the PR, the differences between AC thickness were significant (*p* = 0.043) but not for the SB thickness (*p* = 0.08).

### AC Thickness has Positive Correlations with SB Thickness, Circularity and the CMMC Number

**Fig. 6:**
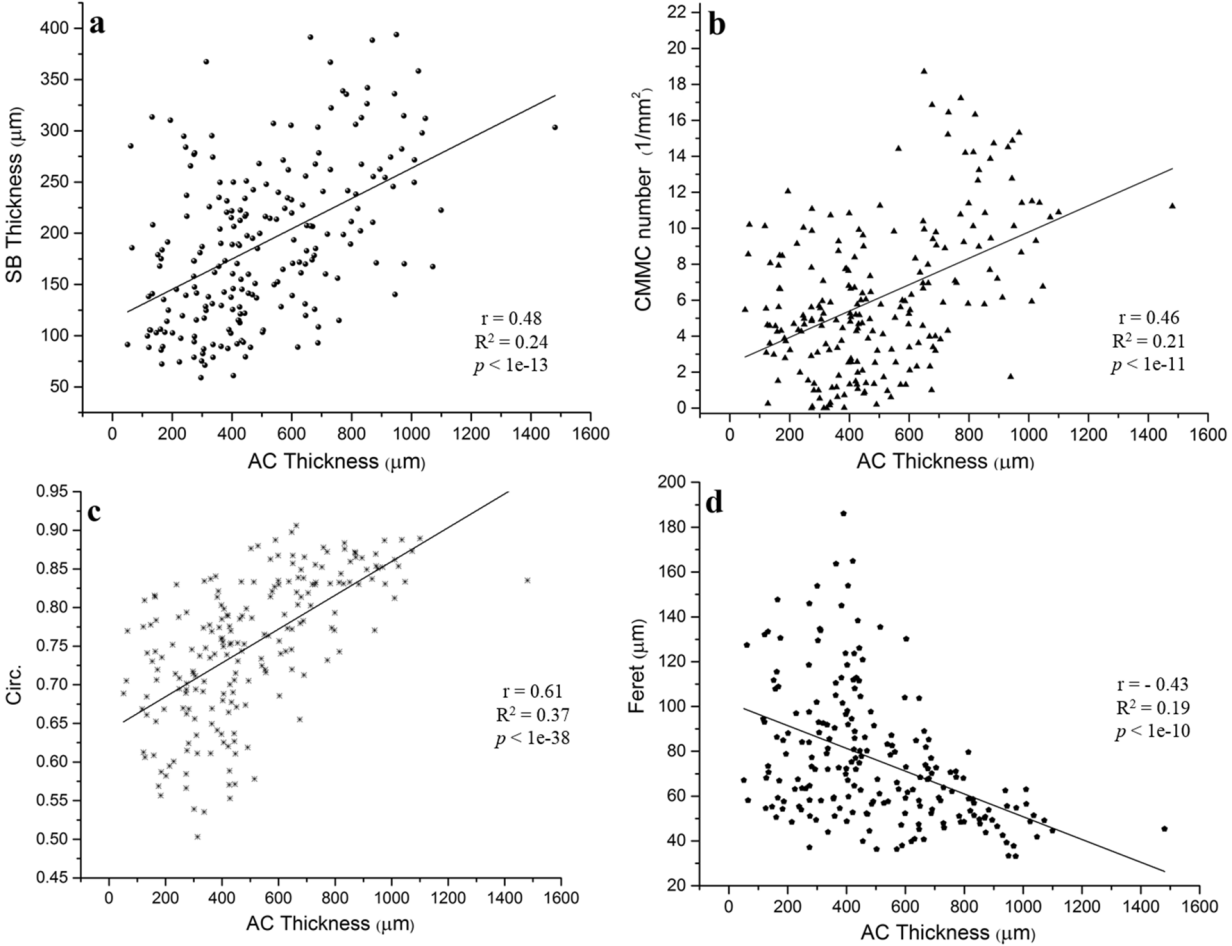
Local correlation of AC thickness with different variables of SB microarchitecture in all the biopsies (*n* = 215) from the five subjects with no signs of osteoarthritis. **a** SB thickness, **b** CMMC number, **c** Circ. and **d** Feret. Each dot in sub-figures represents the corresponding mean value of a measuring point in the uppermost 50 µm of the subchondral bone

For all the biopsies across the entire regions of the femoral head (*n* = 215), it was found that several variables of the subchondral bone and its microarchitecture were correlated with the local AC thickness. SB thickness, CMMC number, and Circ. showed moderate positive correlation with the cartilage thickness, having respective Pearson’s r of 0.48 (*p* < 1e-13), 0.46 (*p* < 1e-11), and 0.61 (*p* < 1e-38) (Fig. 6a–c). The size of the CMMC showed a moderate negative correlation with cartilage thickness (*r *= -0.43; *p* < 1e-10, Fig. 6d).

### Isolated Pores in Histology Slides are Cross-Sections of the CMMC

**Fig. 7:**
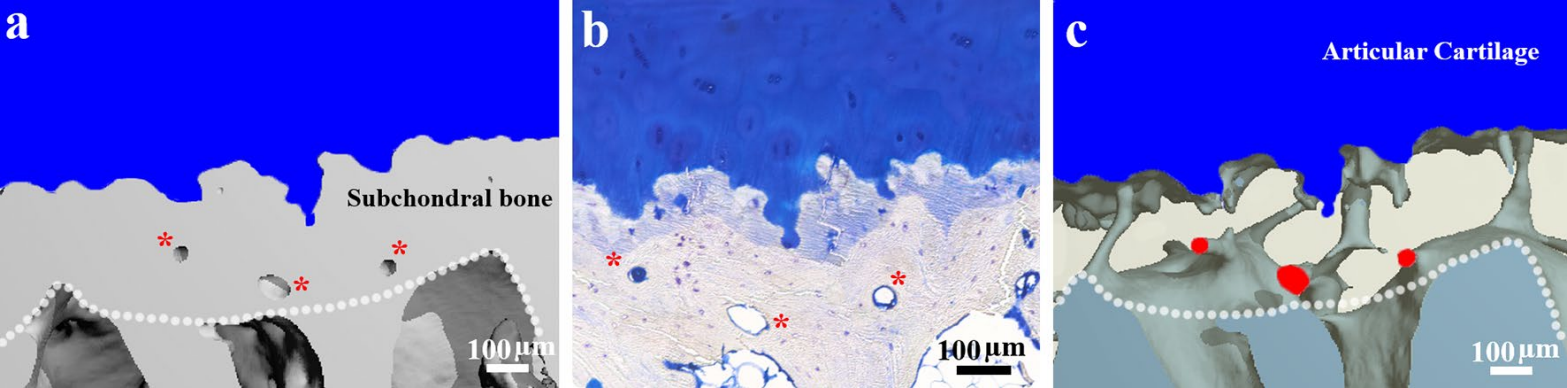
Three representations for the same cross-section of the AC-SB interface. **a** The 3D image showed the subchondral plate and isolated microporous structures (marked by red asterisks). The white dotted line signified the lower margin of the subchondral bone plate. **b** In the Toluidine blue staining the undulating AC-SB interface, the calcified cartilage layer (pastel navy blue), and the identical microporous features are shown. **c** The "negative" image revealed that the previously-observed microporous structures (marked in red) extended to the background, and were in fact part of the CMMC of the SB

To better understand the configuration of the CMMC, the cartilage-subchondral bone interface of an exemplary NLBR sample is represented via positive and negative 3D-reconstructed models, as well as the corresponding toluidine blue stained slide (Fig. 7). Scattered microporous structures were found within the subchondral bone plate of the positive 3D model (Fig. 7a, red asterisks), and were verified when compared to the toluidine blue staining of the same cross-section (Fig. 7b). The negative 3D-reconstructed model, however, showed that these sporadic microfeatures were cross-sections of a continuous microchannel network that might give the impression of being isolated in histology slides (Fig. 7c).

## Discussion

AC and SB interact cooperatively and synergistically through a complex interface, which influences the functionality and the health of the whole joint [6]. Despite this, studies on SB microarchitecture and its potential role as a diagnostic and therapeutic target have been extremely scarce. To the best of the authors' knowledge, in the scientific literature, there is a noticeable gap between the most recent references that have focused on the canalicular connections between AC and SB in the femoral head [14, 17, 21]. Likewise, the 3D representation of the osteochondral junction has so far been reported in only one case, where sequential micrographs of haematoxylin and eosin stainings were overlaid [19]. Here, we created a high-resolution 3D map of the SB in healthy human femoral heads, and identified a complex microchannel network that connects the medullary cavity to the basal cartilage.

In agreement with our study hypothesis, we found distinct characteristics for the CMMC in different regions of human femoral heads with no pre-clinical signs of OA that seemed to correspond with the estimated joint reaction forces on the hip, derived from a separate young healthy cohort. In particular, the local number of the CMMC was significantly higher in the LBR of the joint compared to the NLBR and the PR. This is in line with other reports conducted on healthy human femoral heads and tibial plateau [14–18, 25]. Lane et al. reported a value of ≈ 6.25 channels/mm^2^ for the vascular channels in the superior region of the joint [15], which is smaller compared to our results in the load-bearing region. This could be explained by two points: (1) the number of vascular channels in the calcified cartilage (CC) is not necessarily equivalent to the CMMC number [15, 25]. While confirmed perforation of the cartilage by capillaries does occur, it may be a less recurring event associated with distinctive morphological features [17]. (2) Lane et al. separated the femoral head into two portions that could be considered *generally more stressed* (the entire superior part) or *less stressed* (the entire inferior part), thus were less specific in terms of defining the loading areas, pooling the vascular channels at the superior portion of the peripheral rim with those of the load-bearing region. [15].

The maximum and minimum diameter of the CMMC were also found to follow a location-specific pattern. Generally, the size of the CMMC was smallest at the probable load-bearing areas on the joint, while becoming progressively large with the change of the area from the NLBR to the peripheral rim. This is in accordance with another report on the human tibial plateau [18]. There is approximately a 10–12% difference between the reported mean sizes, which can be due to the size-based categorization of the microchannels in other studies, compared to our region-based classification system [17, 18]. It is also reported that the "pore" sizes on the upper surface of the calcified cartilage in human distal femurs are in the range of 1.98 – 114.2 µm, with the most pores being in the range of 0–30 µm [39]. However, based on our 3D-data the pores below 7 µm are predominantly craters, which are typical of the calcification that normally occurs around chondrocytes [40]. They do not penetrate the SB, are different from the CMMC, and thus were filtered out in the current study. Clearly, pooling these pores with the CMMC would result in an underestimation of the mean size of the microchannels.

The morphology of microchannels is known to exhibit location-specific characteristics, and, thus, has been classified by several investigations [14, 17, 21, 22]. However, the descriptive nature of these studies may be open to interpretation. In this context, the morphology of the CMMC was quantified by their circularity index. We found that the Circ. of the CMMC was progressively reduced as the region shifted from the LBR to the PR, which uniformly expressed the CMMC's "irregularity" in non-central regions. At deeper distances from the tidemark (> 150 µm), the profile of Circ. showed a steep decline irrespective of the loading area, which was reflective of the microchannels’ integration close to the subchondral end-plate, as well as the irregular shape of the trabeculae spacings below the end-plate.

We found a location-dependent gradient of the AC and SB thicknesses, where the regions corresponded to probable load-bearing areas of the joint (Fig. 5). Similar observations are reported for femoral heads, as well as other human joints [14, 17, 18, 41, 42]. The overlap of the AC and SB thickness distributions can be interpreted as an expression of the biomechanical function in the joint and reaffirms the interdependent nature of the cartilage-bone unit [43]. Intriguingly, in areas where the thickest articular cartilage was detected, the CMMC had generally the highest local density. In such regions, the length of the diffusion pathway from the subchondral region to the basal cartilage layer is shorter than that from the cartilage surface [44], which is in favor of the hypothesis that SB perforations are pathways for a SB-driven nutrition of chondrocytes that lie close to the tidemark [40, 45]. It is even asserted that more than 50% of the glucose, oxygen, and water requirements of cartilage can be supported by the perfusion of subchondral vessels [46]. Indeed, a study on adult male baboons showed that long term (> 3 years) interruption of the contact between the subchondral bone and articular cartilage leads to degenerative changes compatible with OA [47]. Hence, unraveling the functionality of the CMMC may have great implications for our understanding of degenerative joint diseases such as OA.

The comparison of the 3D-reconstructed images with the histology slides could explain a recurring dichotomy in the literature (Fig. 7). Since the microchannels are not completely perpendicular in relation to the tidemark, a vertical plane of sectioning would hardly reveal a "complete" CMMC that is connected to the cartilage. Generally, the CMMC appear to be incomplete [14]. Referred to as "virtual islands" [19], these are cross-sections of the CMMC that give the impression of being isolated in 2D micrographs or histology slides (Fig. 7). This may explain why histological and photomicrographical examinations have reported that SB perforations rarely cross the calcified cartilage layer in mature models [11, 23]. Indeed, when the cartilage was severed off down to the CC layer and was examined from a superior angle, several microvessels were found to traverse the CC [15].

There are different definitions in literature, what constitutes a load-bearing region of the femoral head [14, 18, 48, 49]. Following the suggested partitioning from literature with our data still led to remaining large intra-regional variability in the SB microarchitecture. Thus, we opted for a gait analysis-based approach to be able to base the partitioning on different physiological loading areas on the joint. We identified that estimated forces were orientated towards the area superolateral to the intersection between the long axis of the femoral head and the femoral surface being slightly inclined anteriorly (Fig. 2b). The direction of loading in our sample is in agreement with the findings from a previous study, which measured the hip contact force using instrumented hip implants [49].

The distinction of the peripheral rim as a separate regional entity was made by the following rationales: (1) Our visual inspections of the scanned biopsies from such regions showed an apparent enlargement of the SB microchannels compared to the possible LBR, and even the NLBR. Hence, it was suspected that the rim of the joint, close to the femoral neck junction, might have its own distinct CMMC metrics. (2) This notion was strengthened when the superimposed reaction forces were viewed at matched angles compared to a schematic model (Fig. 2). The forces were visibly clustered on the superocentral portion of the joint, excluding the rim. Interestingly, it is known that the shear stress concentration is particularly high at the margins and peripheral areas of the joint [50], and that subchondral bone plate has less density compared to the weight-bearing regions [51]. In line with our study hypothesis regarding the interrelationship of CMMC characteristics with possible loading areas on the joint, it was then conceivable that the PR might be a separate entity. (3) There are two reports that in fact acknowledge the differences in the SB microstructure between the weight-bearing regions and the margins close to the femoral neck junction [14, 18]. Therefore, the peripheral rim of the joint was recognized as a separate identified region for categorization of the computed CMMC metrics.

Limitations of this study include: (1). The small sample size (*n *= 5) and the unknown medical history of the human cadavers. Moreover, the sex and the age of the cadavers were initially unknown, and were determined postmortem (Online Resources 1 and 2). (2) The measurement of the CMMC characteristics in sequential 2D layers rather than directly in 3D, due to the big sizes of the reconstructed models and long processing times (By extrapolation and according to Perilli et al. [52], it is estimated that the 3D computation time for each cartilage-bone cylinder would have taken 12 h using a desktop computer equipped with a quad-core Intel® Xeon® Processor E5-1620 v4 running at 3.5 GHz, 32 GB memory, OS Windows 7, 64-bit, available at the Department of Trauma Surgery, Orthopaedic Surgery and Plastic Surgery, University Medical Center Göttingen). For this reason, Feret. and Circ. can also reflect the angle at which the microchannels run through the SB, as diagonal channels are cut by a transverse plane of sectioning, which can possibly lead to an overestimation of the values. Nevertheless, at the uppermost 50 µm of the SB the channels are perpendicularly connected to the AC-SB interface, minimizing this potential effect. (3) The discrete sampling (43 measuring points from each joint), which has limitations in describing the continuous nature of the joint. (4) The difference between the age and sex of our participants in the gait experiments (young men) and the donors of the cadaveric samples (older females) owing to the availability of the subjects. These two populations may exhibit slightly different loading of the femoral head during walking. Previous studies on level ground walking have reported that, compared to men, women exhibit greater pelvic list, adduction and external rotation of the hip during early stance, and internal rotation of the hip between mid to late stance, all by approximately 2 to 4 degrees on average [53–56]. As for the effects of age, previous studies have reported that, older individuals exhibit increased hip flexion during early stance (by approximately 2 to 5 degrees), decreased hip extension between mid to late stance (by approximately 5 to 7 degrees), and reduced hip adduction during early stance (by approximately 3 degrees) [57–59]. These differences in the relative rotations of the pelvis and femur can affect where the femoral head is loaded during walking. However, given their small magnitudes as well as our definition of the LBR, which conservatively encompasses the estimated area of loading (Fig. 2), these sex- and age-related discrepancies are unlikely to affect our results. Furthermore, the aforementioned studies on aging compared individuals in their third decade of life to those in their eighth to ninth decade of life [57–59]. Because our cadaveric samples were aged approximately between fifth and sixth decades of life, we speculate that the age-related effects would be less than the reported values. (5) We estimated how the hip reaction force was oriented and not how the force was distributed over the femoral head. In reality, areas larger than what is shown in Fig. 2b would be loaded during walking. Future studies should incorporate anatomically-faithful modeling of the hip joint (e.g., [60]) to investigate how the load is distributed over the femoral head and how the distribution compares to the microchannel architecture inside the subchondral bone of the femoral head.

In conclusion, we have profiled and quantified the 3D cartilage-bone marrow microchannel connectors of the SB in cadaveric femoral heads with no signs of OA and its association with typical local forces on the joint derived from a healthy cohort. Generally, the CMMC are abundant, small, and circular in areas that are probably load-bearing, while sporadic, significantly larger, and irregularly-shaped in the non-load-bearing region and the peripheral rim of the femoral head. The AC thickness shows a significant moderate positive correlation with the CMMC number, the circularity of the microchannels and the SB thickness, while exhibiting a moderate negative correlation with the size of the CMMC. Since there is no consensus on the precise functional purposes of these CMMC, more investigations are necessary in order to uncover their potential role in joint pathophysiology.

## Supplementary Information

Below is the link to the electronic supplementary material.

Supplementary file1 (DOCX 231 KB)

Supplementary file2 (DOCX 4546 KB)

## Data Availability

The data that support the findings of this study are available from the corresponding author (AFS) upon reasonable request.

## Abbreviations

AC: Articular cartilage
SB: Subchondral bone
CMMC: Cartilage-bone marrow microchannel connectors
LBR: Load-bearing region
NLBR: Non-load-bearing region
PR: Peripheral rim
OA: Osteoarthritis
CC: Calcified cartilage
MHH: Medizinische Hochschule Hannover
Micro-CT: Microcomputed Tomography
Circ: Circularity index
Feret: Maximum caliper dimeter
MinFeret: Minimum caliper dimeter
ANOVA: One-way analysis of variance
