# Supplementary material for: Investigating the Microchannel Architectures Inside the Subchondral Bone in Relation to Estimated Hip Reaction Forces on the Human Femoral Head
Source: Calcif Tissue Int. 2021 May 22;109(5):510–24. doi: 10.1007/s00223-021-00864-x (PMC8484212; doi:10.1007/s00223-021-00864-x)
Supplement: Supplementary file 1 — Supplementary file1 (DOCX 231 KB) [file 223_2021_864_MOESM1_ESM.docx]

**Online Resource 1. Experimental method: Sex determination**

in

The contact force of the human femoral head correlates with the microchannel architecture inside the subchondral bone

Shahed Taheri^1^, Takashi Yoshida^1^, Kai O. Böker^1^, Robert H. Foerster^1^, Lina Jochim^1^, Anna Lena Flux^2^, Birgit Grosskopf^2^, Wolfgang Lehmann^1^, Arndt Friedrich Schilling^*1^

^1^Department of Trauma Surgery, Orthopaedic Surgery and Plastic Surgery, University Medical Center Göttingen, Göttingen, Germany;

^2^University of Göttingen Johann-Friedrich-Blumenbach-Institute for Zoology & Anthropology, Department of Historical Anthropology and Human Ecology, Göttingen, Germany;

**^*^** Corresponding author: [arndt.schilling@med.uni-goettingen.de](mailto:arndt.schilling@med.uni-goettingen.de)

**1.1 DNA extraction**

Since the quality of DNA in formalin-fixed tissues is poor, modified extraction methods should be applied to yield long-fragmented quality DNAs. Hence, a heat-assisted breakage of the DNA-protein cross-links was used based on the protocol of Campos and Gilbert [29]. Briefly, the fixed tissue powder was incubated in an alkali buffer solution (0.1M NaOH with 1% SDS), and was heated with the aid of an autoclave to 120°C for 25 min. Nucleic acids were then removed from cross-linked proteins by a 25:24:1 phenol:chloroform:isoamyl alcohol solution, and were further purified in subsequent steps.

Afterwards, DNA concentration was measured by DeNovix DS-11 FX+ spectrophotometer (Wilmington, USA). The absorbance ratios (1.8 < A_260_/A_280/230_ < 2.0) indicate high quality DNA. Samples were stored at −20°C until further processing.

**Online Table 1.** Quality characteristics of the extracted DNA

| Subjects | Concentration (ng/µL) | A_260_/A_280_ | A_260_/A_280_ Alert | A_260_/A_230_ | A_260_/A_230_ Alert |
| --- | --- | --- | --- | --- | --- |
| 1 | 377.28 | 1.84 | Met criteria | 1.83 | Met criteria |
| 2 | 432.08 | 1.9 | Met criteria | 2.03 | Met criteria |
| 3 | 1376.061 | 1.94 | Met criteria | 2.01 | Met criteria |
| 4 | 108.214 | 1.92 | Met criteria | 2.14 | Met criteria |
| 5 | 993.7 | 1.96 | Met criteria | 2.28 | Met criteria |

**1.2 PCR-based sex identification**

To determine the sex of the individuals, a polymerase chain reaction (PCR)-based gonosomal sexing system (C. Roos 2010, unpublished data) was used. Primers were designed to generate a specific product at the X-chromosome (163 bp) and Y chromosome (137 bp). Sequences are available from the authors upon request.

PCR was conducted in 50 µl of master mixture, containing 100 ng of DNA solution, 0.5 µl of each dNTP (Biozym, Hessisch Oldendorf, Germany), 2 µl of each forward and reverse primers, 0.25 µl Taq DNA Polymerase (Biozym, Hessisch Oldendorf, Germany), 5 µl Buffer (Biozym, Hessisch Oldendorf, Germany) and 39.25 µl sterile ultrapure water. Amplification was performed on a thermal cycler (SensoQuest Labcycler, Göttingen, Germany) using a programme consisted of 5 min denaturation at 95°C, followed by 40 cycles of denaturation (95°C, 30 sec), annealing (56 °C, 30 sec) and primer extension (72 °C, 30 sec). The final cycle was followed by extension at 72 °C for 3 mins and indefinite hold time at 4 °C. The PCR products were separated on 2% agarose gel at 120V for 45 min and stained with GelRed [49]. The products were viewed using UV illumination and documented.

The validity of the PCR amplification method was tested with two positive controls (one male and one female), which were extracted from fresh-frozen samples of human tissues. As expected, male control (Ctrl M) yielded two bands (163bp and 137 bp), whereas female control (Ctrl F) one band only (163 bp). **Online Figure 1** shows the sex identification of the five femurs used in this study (Subjects 1-5), where all of them revealed female-specific bands. The negative control (NC) showed no signal and, therefore, consisted only of master mix and no DNA contamination. Our results show that the applied amplification method is highly specific since no miss-amplification was observed and no amplification occurred in the absence of DNA (negative control).





**Online Figure 1.** A PCR-based sex determination method. Marker indicated the 100-bp ladder marker. The lanes 2-6 correspond to the human femurs tested for sex identification. The lanes 7 and 8 demonstrate the positive female (Ctrl F), and male control (Ctrl M), respectively. The negative control (NC) which is consisted only of mater mix and no DNA contamination is shown in lane 10.

**1.3 References**

29. Campos, P.F.; Gilbert, T.M.P. DNA Extraction from Formalin-Fixed Material. In *Methods in molecular biology (Clifton, N.J.)*; 2012; Vol. 840, pp. 81–85.

49. Crisafuli, F.A.P.; Ramos, E.B.; Rocha, M.S. Characterizing the interaction between DNA and GelRed fluorescent stain. *Eur. Biophys. J.* **2015**, *44*, 1–7, doi:10.1007/s00249-014-0995-4.
