# Supplementary material for: Investigating the Microchannel Architectures Inside the Subchondral Bone in Relation to Estimated Hip Reaction Forces on the Human Femoral Head
Source: Calcif Tissue Int. 2021 May 22;109(5):510–24. doi: 10.1007/s00223-021-00864-x (PMC8484212; doi:10.1007/s00223-021-00864-x)
Supplement: Supplementary file 2 — Supplementary file2 (DOCX 4546 KB) [file 223_2021_864_MOESM2_ESM.docx]

**Online Resource 2. Microscopic investigation: Determination of the biological age**

in

The contact force of the human femoral head correlates with the microchannel architecture inside the subchondral bone

Shahed Taheri^1^, Takashi Yoshida^1^, Kai O. Böker^1^, Robert H. Foerster^1^, Lina Jochim^1^, Anna Lena Flux^2^, Birgit Grosskopf^2^, Wolfgang Lehmann^1^, Arndt Friedrich Schilling^*1^

^1^Department of Trauma Surgery, Orthopaedic Surgery and Plastic Surgery, University Medical Center Göttingen, Göttingen, Germany;

^2^University of Göttingen Johann-Friedrich-Blumenbach-Institute for Zoology & Anthropology, Department of Historical Anthropology and Human Ecology, Göttingen, Germany;

**^*^** Corresponding author: [arndt.schilling@med.uni-goettingen.de](mailto:arndt.schilling@med.uni-goettingen.de)

**2.1 Background**

When investigating archaeological or forensic skeletons, there are several anthropological methods to determine the age at death. In subadults the skeleton undergoes age-dependent changes in growth and maturation, making it relatively easy to determine the biological age. In adults, the age-dependent changes are mainly degenerative alterations on the skeleton, which are highly affected by the living conditions of the individual (health, nutritional conditions, or physical stress) and, therefore, the morphological age determination is aggravated and can differ from the calendrical age. The calendrical age can be determined by the microscopic investigation of the incremental lines in the tooth cementum (e.g. [50,51]). However, morphological methods for the age determination of an individual are highly dependent on the state of preservation of the skeleton and are not applicable for the age determination of isolated bones. In this case, another microscopic method can be applied to access the age-dependent changes in the cortical bones’ microstructure in cross-sections of human long bones.

In general, the cortical bone shows several structural elements like primary and secondary osteons, haversian canals, endosteal and periosteal lamellar bone, and areas of bone resorption, whose quantity or proportion undergo age-dependent changes. These gradual changes in the microstructure of different bones can be assessed quantitatively and the biological age can be calculated by the use of a regression formula (e.g. [52–55]). The precision of these quantitative methods is aggravated when the microscopic picture of the investigated bone material, especially in archaeological samples, shows degradation of the bone surface or destruction through the activity of microorganisms. Furthermore, the bones’ microstructure can be altered due to e.g. variable exposure to physical stress in different areas of the investigated cross section [56]. Hence, it is mandatory to quantitatively assess various areas of the cross-section of the bone, making it a time-consuming method. Alternatively, the microstructure of the cortical bone can be assessed qualitatively. The different manifestations of the structural elements mentioned above vary in different age-groups (Online Figure 2) [57] and can be divided into adult (20-40 years), mature (40-60 years), and senile (60+ years). Often, the age-groups “adult” and “mature” can be further divided into three sub-groups (early, middle, and late) to narrow down the biological age.


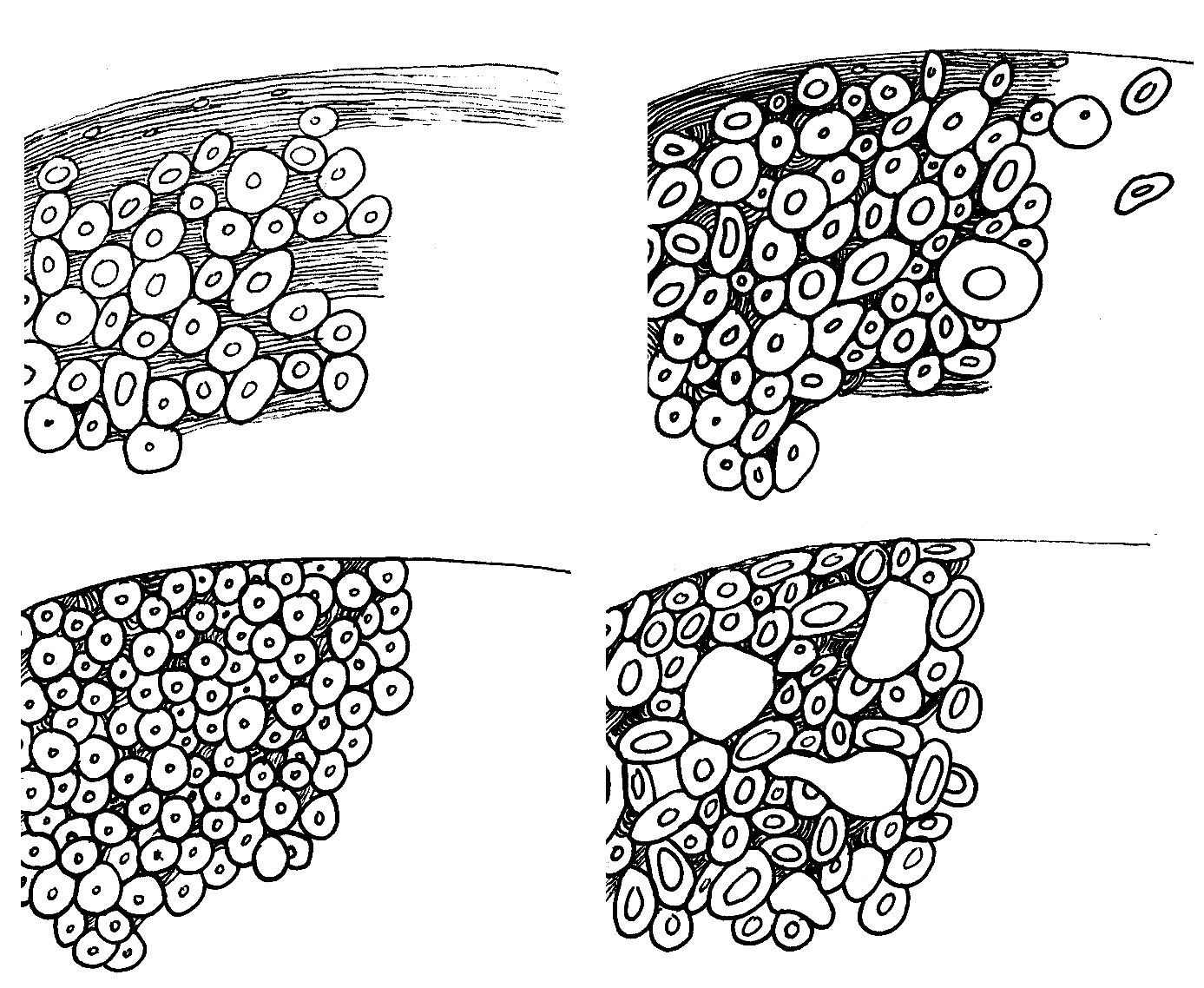


**d)**

**c)**

**b)**

**a)**

**Online Figure 2.** Schematic representation of the different manifestations and alterations of the structural elements in the microstructure of the cortical bone (femur) across the age groups juvenile (a), adult (b), mature (c), and senile (d) [57].

**2.2 Methods**

Cortical bone samples from the midshaft of the anterior part of the femur diaphysis were sawed out. The bones were briefly macerated according to the following protocol: they were rinsed in an ultrasonic bath fillet with distilled water for one hour to remove the excess formalin solution (the distilled water was renewed every 15 minutes). Afterwards, the bone samples were incubated overnight in a 10% NaCl/detergent solution to remove fatty compounds. The solution was renewed and cooked for 4 hours to remove the periosteum. The bone samples were transferred to a renewed NaCl/detergent solution and incubated for 36 hours at 50°C. Afterwards, they were rinsed again in an ultrasonic bath fillet with distilled water for 3 hours (the distilled water was renewed every 30 minutes). The samples were then dehydrated in an ethanol series with ascending concentrations (50%, 70%, 80%, 96%, 99.9% abs.) for 15 minutes each, transferred to acetone, and dried overnight at 50°C. Afterwards, every sample was embedded separately in the epoxy resin Biodur® [58] under vacuum, allowing the resin to impregnate the very fine canaliculi of the bone, which reduced optical artefacts to a minimum during microscopic investigations [57]. After the polymerization of the Biodur® (approx. 72 hours) thin cross sections (approx. 100µm) were prepared by using a saw microtome (Leica SP 1600). The bones’ microstructure was microscopically examined with plain and polarization light in different magnifications.

**2.3 Results**

It was possible to determine the biological age for four of the investigated subjects (Online Table 2). Several areas in every cross section were examined and the different structural elements mentioned above were assessed qualitatively.

**Online Table 2.** Age determination for the investigated bones

| Investigated bone | Determined biological age |
| --- | --- |
| Subject 1 | early mature (40 to mid-40) |
| Subject 2 | late mature (mid-50 to 60) |
| Subject 3 | early mature (40 to mid-40) |
| Subject 4 | older than subadult |
| Subject 5 | early mature (40 to mid-40) |

As an example, the microscopic picture of one bone (Subject 5), which was determined as early mature, is illustrated in Online Figure 2. In one cross section (Subject 3), the determined biological age varied between the different investigated areas of the cross-section. The microstructure of the bone was consistent with the early to middle mature age group in the majority of investigated areas. In contrast, some areas showed several lamellar structures which are typical characteristics for the adult age class and uncommon in older individuals. However, it is known that these lamellar alterations can be found in individuals suffering from atrophy in the respective bone [59] and, therefore, for the age determination of the individual (Subject 3) these areas with lamellar structures were excluded. For one of the investigated bones (Subject 4) the age could not be determined more exact than older than subadult. The microstructure of the bone showed very unusual alterations with predominantly lamellar bone and only a few osteons on the one side of the bone (typical characteristics for a subadult individual) and on the other side structural elements indicating a much older individual. A closer look to the bone itself demonstrated a significant lower thickness of the cortical bone compared to the others, indicating some sort of alterations as a consequence of e.g. a well healed trauma.


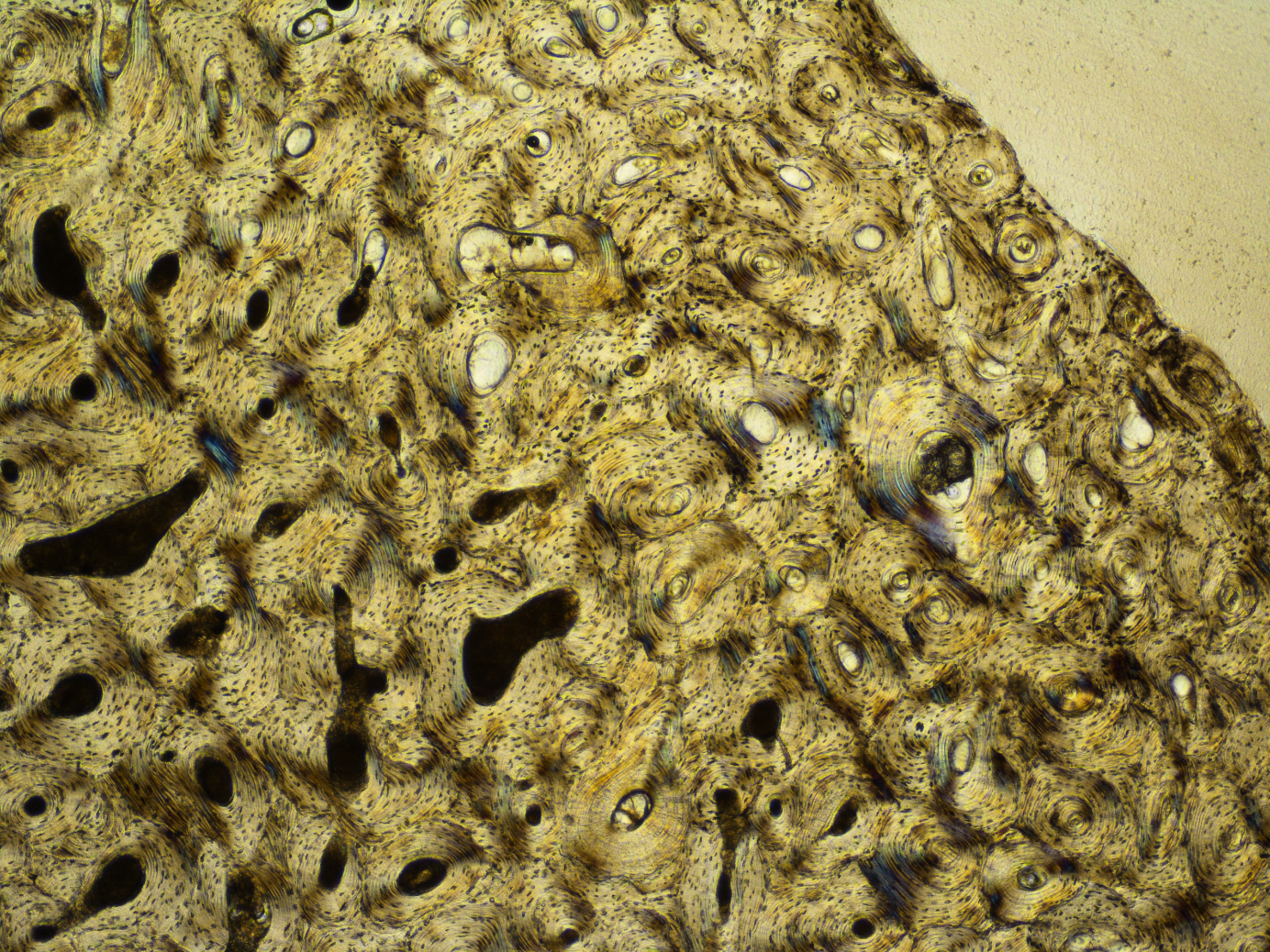


**Online Figure 3.** The microscopic picture of Subject 5 (plain light with little polarization filter, 40x magnification). The microstructure reveals predominantly irregularly formed osteons, which replaced the periosteal lamellar bone almost entirely. The areas of bone resorption are limited, indicating the individual died in the early mature age group.

**2.4 References**

50. Grosskopf, B. Individualaltersbestimmung mit Hilfe von Zuwachsringen im Zement bodengelagerter menschlicher Zaehne. *Zeitschrift fur Rechtsmedizin* **1990**, *103*, 351–359, doi:10.1007/BF00204456.

51. Grosskopf, B.; McGlynn, G. Age diagnosis based on incremental lines in dental cementum: A critical reflection. *Anthropol. Anzeiger* **2011**, *68*, 275–289, doi:10.1127/0003-5548/2011/0004.

52. Kerley, E.R. The microscopic determination of age in human bone. *Am. J. Phys. Anthropol.* **1965**, *23*, 149–163, doi:10.1002/ajpa.1330230215.

53. Kerley, E.R.; Ubelaker, D.H. Revisions in the microscopic method of estimating age at death in human cortical bone. *Am. J. Phys. Anthropol.* **1978**, *49*, 545–546, doi:10.1002/ajpa.1330490414.

54. Uytterschaut, H. Human Bone Remodelling and Aging. In *Histology of Ancient Human Bone: Methods and Diagnosis*; Springer Berlin Heidelberg: Berlin, Heidelberg, 1993; pp. 95–109.

55. Stout, S.D.; Stanley, S.C. Percent osteonal bone versus osteon counts: The variable of choice for estimating age at death. *Am. J. Phys. Anthropol.* **1991**, *86*, 515–519, doi:10.1002/ajpa.1330860407.

56. Andrea Drusini Refinements of two methods for the histomorphometric determination of age in human bone. *Z. Morphol. Anthropol.* **1987**, *77*, 167–176.

57. Grosskopf, B. Leichenbrand - Biologisches und kulturhistorisches Quellenmaterial zur Rekonstruktion vor- und frühgeschichtlicher Populationen und ihrer Funeralpraktiken 2004.

58. Hagens, G. Von Impregnation of soft biological specimens with thermosetting resins and elastomers. *Anat. Rec.* **1979**, *194*, 247–255, doi:10.1002/ar.1091940206.

59. Herrmann, B.; Grupe, G.; Hummel, S.; Piepenbrink, H.; Schutkowski, H. Labormethoden. In *Prähistorische Anthropologie*; Springer Berlin Heidelberg: Berlin, Heidelberg, 1990; pp. 46–282.
